# Usp5, Usp34, and Otu1 deubiquitylases mediate DNA repair in *Drosophila melanogaster*

**DOI:** 10.1038/s41598-022-09703-x

**Published:** 2022-04-07

**Authors:** Zoltán G. Páhi, Levente Kovács, Diána Szűcs, Barbara N. Borsos, Péter Deák, Tibor Pankotai

**Affiliations:** 1 Institute of Pathology, Albert Szent-Györgyi Medical School, University of Szeged, 1 Állomás Street, 6725 Szeged, Hungary; 2 Department of Genetics, Faculty of Sciences and Informatics, University of Szeged, 52 Közép fasor, 6726 Szeged, Hungary; 3 Divison of Biology and Biological Engineering, California Institute of Tehnology, 1200 East California Boulevard, Pasadena, 91125 USA

**Keywords:** Biological techniques, Cell biology, Developmental biology, Genetics

## Abstract

Ubiquitylation is critical for preventing aberrant DNA repair and for efficient maintenance of genome stability. As deubiquitylases (DUBs) counteract ubiquitylation, they must have a great influence on many biological processes, including DNA damage response. To elucidate the role of DUBs in DNA repair in *Drosophila melanogaster*, systematic siRNA screening was applied to identify DUBs with a reduced survival rate following exposure to ultraviolet and X-ray radiations. As a secondary validation, we applied the direct repeat (DR)-white reporter system with which we induced site-specific DSBs and affirmed the importance of the DUBs Ovarian tumor domain-containing deubiquitinating enzyme 1 (Otu1), Ubiquitin carboxyl-terminal hydrolase 5 (Usp5), and Ubiquitin carboxyl-terminal hydrolase 34 (Usp34) in DSB repair pathways using Drosophila. Our results indicate that the loss of *Otu1* and *Usp5* induces strong position effect variegation in Drosophila eye following I-SceI-induced DSB deployment. Otu1 and Usp5 are essential in DNA damage-induced cellular response, and both DUBs are required for the fine-tuned regulation of the non-homologous end joining pathway. Furthermore, the Drosophila DR-white assay demonstrated that homologous recombination does not occur in the absence of Usp34, indicating an indispensable role of Usp34 in this process.

## Introduction

DNA damage-induced cellular response is a primary mechanism to protect cells against cancerous malformation. To maintain genomic integrity, this process is critical for the recruitment and activation of specific proteins, including writers, readers, and erasers of the epigenetic marks, implicated in the DNA repair-related rearrangement of the chromatin structure. These posttranslational modifications (PTMs) contribute to the fine-tuned regulation of the proteins involved in the DNA damage response (DDR) and the subsequent repair processes^1–3^. These modifications are also critical for the appropriate recruitment of downstream repair proteins to modulate the effectiveness and dynamics of DNA repair^4^.

Among these PTMs, ubiquitylation is one of the well described mechanisms, involving a cascade of activating, conjugating, and ligating enzymes. The ubiquitin enzyme cascade catalyzes the formation of an isopeptide bond between the C-terminal glycine (G) residue of the ubiquitin molecule and lysine (K) residue(s) of the target proteins. Removal of ubiquitin groups is catalyzed by deubiquitylases (DUBs), and the mechanism has received increasing attention for understanding of their importance in DNA repair^5,6^. In recent years, several DUBs have already been described to participate in DNA repair. In U2OS cells, human ubiquitin-specific peptidase 1 (USP1) was demonstrated to be essential to promote homologous recombination (HR)^7,8^. Furthermore, human ubiquitin-specific peptidase 3 (USP3) is implicated in the deubiquitylation of histone H2A/H2B and is required for the efficient resolution of replication-coupled stress conditions^9^. In addition, in human cells, USP4, USP5, USP7, and USP28  were shown to be involved in controlling the stability of p53 during DNA repair^10–13^. USP20 regulates the Ataxia telangiectasia and Rad3-related protein (ATR)-related DDR, whereas USP7 and USP47 facilitate Ataxia telangiectasia mutated (ATM)-related DDR^13–15^. USP7 together with USP34 stabilizes Ring Finger Protein 168 (RNF168) implicated in H2A monoubiquitylation^16,17^. Moreover, the human ovarian tumor domain-containing protein 4 (OTUD4) is implicated in the regulation of DNA repair through an ATM/p53-mediated pathway^18^, whereas OTU Deubiquitinase, OTU domain-containing ubiquitin aldehyde-binding protein 1 (OTUB1) and OTU Deubiquitinase, OTU domain-containing ubiquitin aldehyde-binding protein 2 (OTUB2) are essential for the DNA damage-induced foci formation^19–21^. On the basis of the expanding knowledge on the DNA damage-induced ubiquitylation process, different ubiquitin linkages have major impacts on the choice of the appropriate repair mechanism. Interconnection between DUBs and other PTMs has been also revealed. For instance, studies have elucidated the crosstalk between poly(ADP-ribosylation) polymerases and DUBs, but its exact mechanism still remains unclear ^22,23^. These data emphasize the importance of the characterization of DUBs which have been reported to be involved in DNA repair.

For elucidating the function of these enzymes and identifying new DUB players implicated in DNA repair, ultraviolet (UV) and X-ray irradiation-based siRNA screenings were applied to identify novel DUBs that may be recruited to DNA lesions. We identified five DUBs (Usp14, Usp34, Usp45, Usp47, and Otu1), which exhibit synthetic lethality following UV irradiation, of those we tested. In addition, after X-ray irradiation, the absence of Usp5 and Otu1 caused more defects in the imaginal disc development of Drosophila than those found in the wild-type strain, suggesting the regulatory roles of Otu1 and Usp5 in the ionizing irradiation-induced repair pathways. In a secondary approach, we applied a DSB repair reporter assay which generates I-SceI endonuclease-induced DSBs in euchromatic and heterochromatic regions. During these analyses, we affirmed the importance of Usp34 DUB in DSB repair pathways in Drosophila and we further determined the effect of these DUBs. These findings provide novel insights into the functional link between DUBs and DNA repair.

## Results

### DUBs are implicated in the regulation of UV-induced DNA repair

**Figure 1:**
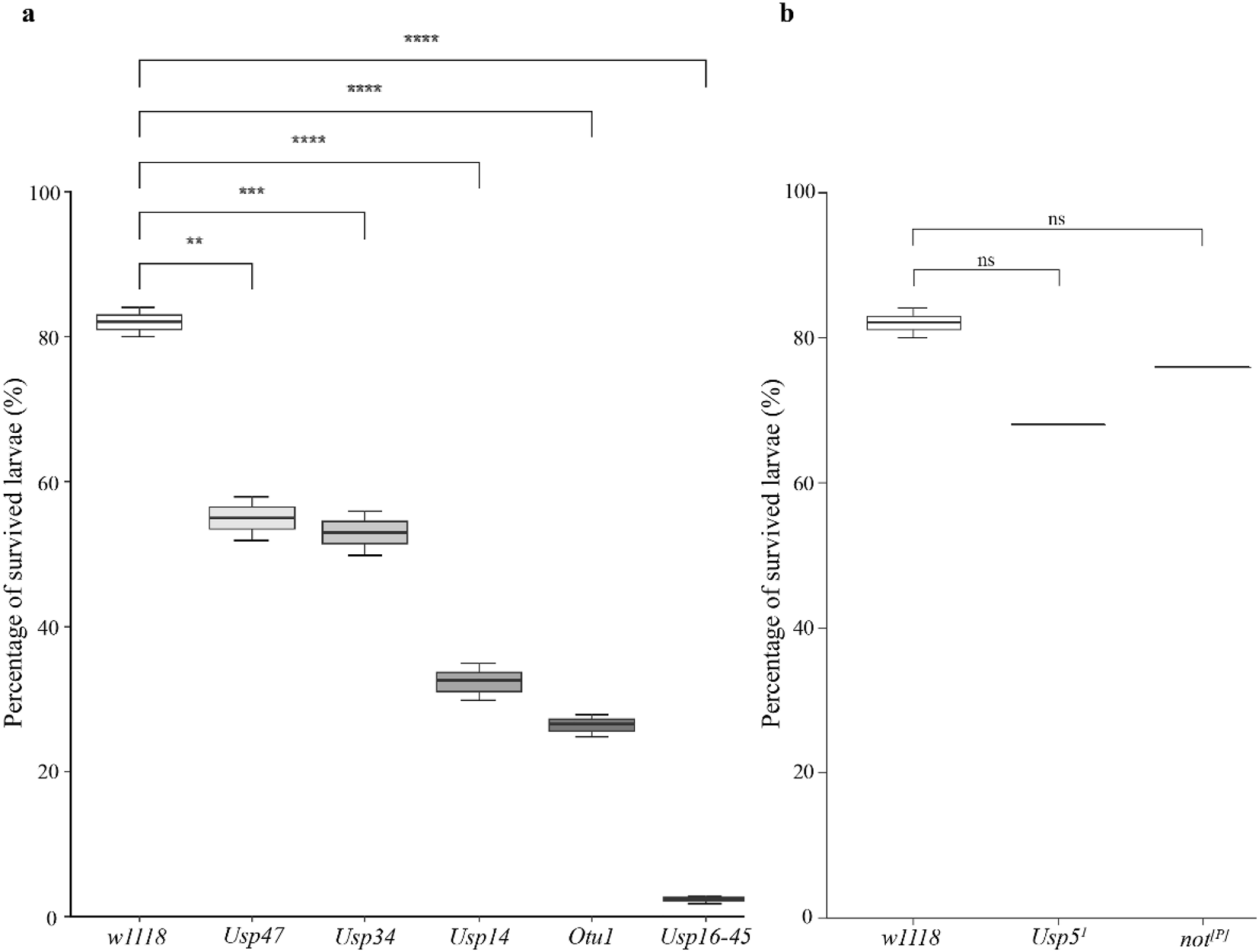
Survival rate of DUB mutant L3 larvae in response to UV irradiation. (**a**) The mean values of the survival rate of the UV-irradiated DUB mutant L3 Drosophila larvae are represented. L3 larvae were exposed to 15 mJ/cm^2^ UV. (**b**) Heterozygous Usp5^1^/TM6b GFP and not^[P]^/TM6b GFP DUB mutant, L3 larvae were irradiated with 15 mJ/cm^2^ UV. For the statistical test, one-way ANOVA in combination with Tukey's post-hoc test was used. Significant values are indicated as follows: **P < 0.01, ***P < 0.001, ****P < 0.0001. Data were obtained from three independent experiments.

For identifying DUBs in *Drosophila melanogaster*, we applied bioinformatics homology search analysis using the sequences of known yeast and human DUBs^24,25^. On the basis of our search results, we identified 44 DUBs that were classified into the following DUB subgroups: 23 Ubiquitin specific proteases (USPs), 4 ubiquitin C-terminal hydrolases (UCHs), 1 Machado-Joseph Disease (MJD), 7 ovarian tumour deubiquitinases (OTUs), and 9 JAB1/MPN/Mov34 metalloenzymes (JAMMs) (Table 1). To identify the putative DUBs that might be implicated in DNA repair, we conducted a DUB siRNA screening and, in some cases, such as *Otu1, Usp5* and *not*, we also used Drosophila null mutant stocks. We used individual *D. melanogaster* transgenic lines, which enabled the siRNA silencing of the 44 different DUBs by the daughterless-galactose-responsive transcription factor (*da-GAL4*) driver (Table 1). As the readout of the screening, we evaluated the viability of L3 larvae and their pupation rate after exposure to UV and X-ray irradiation. We first investigated the sensitivity of DUB siRNA and null mutant Drosophila L3 larvae following exposure to 15 mJ/cm^2^ UV. Then, the irradiated larvae were collected and maintained for 24 h on a standard medium, and the survived larvae were counted (Supplementary Fig. 1). Of the DUBs we tested, *usp47* (*CG5486*), *usp34* (*CG5794*), *usp14* (*CG5384*), *Otu1* (*CG4603*), and *usp16–45* (*CG4165*) DUB mutant Drosophila stocks demonstrated a significant difference in viability compared with the control (*w1118*). The most significant difference was measured in the case of *Usp16–45*, *Usp14* siRNA-silencing *D. melanogaster* strains and *Otu1*^*1*^ (w^+^;+;*CG4603*^*Δ101*/2^/*CG4603*^*Δ101*/2^) Drosophila null mutants, whereas the suppression of *Usp34* and *Usp47* resulted in milder, but still significant difference compared with those of the control (Fig. 1a). This experiment indicated that several DUBs play an indispensable role in the UV-induced DNA repair pathways.

**Table 1:** A 44 Drosophila DUBs were selected for further tests in the RNAi screening.

FlyBase symbol and annotations symbols	Human ortholog symbol	DUB class	Stock centre and Stock ID	Functional domains
*CSN5 (CG14884)*	COPS5 (85%)	JAMM	Bloom: JF03159	JAMM
*Rpn11 (CG18174)*	PSMD14 (93%)	JAMM	Bloom: HMS00071	JAMM; Mit-Mem
*CG2224*	AMSH (61%)	JAMM	VDRC: 108622	JAMM; MIT
*CG4751*	MPND (17%)	JAMM	VDRC: 45530	JAMM
*CSN6 (CG6932)*	COPS6 (75%)	JAMM	VDRC: 105385	JAMM
*eIF3f2 (CG8335)*	EIF3F (45%)	JAMM	VDRC: 15506	JAMM; Mit-Mem
*Prp8 (CG8877)*	PRPF8 (94%)	JAMM	VDRC: 18567	JAMM; RNaseH
*eIF3h (CG9124)*	EIF3H (61%)	JAMM	VDRC: 106189	JAMM
*eIF3f1 (CG9769)*	EIF3F (52%)	JAMM	VDRC: 101465	JAMM; Mit-Mem
*CG3781*	JOSD1 (65%), JOSD2 (64%)	MJD	VDRC: 108379	Josephin
*otu (CG12743)*	OTUD4 (30%)	OTU	NIG-Fly: 12743R-3	OTU; Tudor
*CG3251*	HIN1-like (38%)	OTU	VDRC: 34574	OTU
*Otu1 (CG4603)*^1^*	YOD1 (49%)	OTU	VDRC: 21894	OTU; UBX; ZnF-C2H2
*CG4968*	OTUB1 (69%)	OTU	VDRC: 21978	OTU
*Duba (CG6091)*	OTUD5 (39%)	OTU	VDRC: 109912	OTU; UIM
*CG7857*	OTUD6B (55%)	OTU	VDRC: 105469	OTU
*trbd (CG9448)*	TRABID (56%)	OTU	VDRC: 24030	OTU; ZnF-RANBP2; ANK
*Uch-L5R (CG1950)*	UCH-L5 (67%)	UCH	NIG-Fly: 1950R-2	UCH
*Uch-L5 (CG3431)*	UCH-L5 (75%)	UCH	VDRC: 32443	UCH
*Uch (CG4265)*	UCH-L3 (67%)	UCH	VDRC: 26469	UCH
*calypso (CG8445)*	BAP1 (36%)	UCH	VDRC: 28904	UCH, Coiled domain
Usp5 (CG12082)*	USP5 (66%)	USP	VDRC: 17567	USP; UBP; UBA
*Usp2 (CG14619)*	USP2 (31%)	USP	VDRC: 37930	USP
*Usp7 (CG1490)*	USP7 (66%)	USP	VDRC: 110324	USP; MATH; ICP0
*Usp1 (CG15817)*	USP1 (29%)	USP	Bloom: JF02992	USP
*faf (CG1945)*	USP9X (64%)	USP	VDRC: 107716	USP
*ec (CG2904)*	USP54 (32%)	USP	VDRC: 106671	USP
*Usp30 (CG3016)*	USP30 (36%)	USP	VDRC: 3246	USP; Transmembrane
*Usp15–31 (CG30421)*	USP31 (37%)	USP	VDRC: 103553	USP; UBL
*Usp10 (CG32479)*	USP10 (44%)	USP	VDRC: 37859	USP
*Usp16–45 (CG4165)*	USP45 (38%)	USP	VDRC: 110286	USP; ZnF-UBP
not (CG4166)*	USP22 (64%)	USP	VDRC: 45775	USP; ZnF-UBP
*Usp14 (CG5384)*	USP14 (71%)	USP	VDRC: 110227	USP; UBL
*Usp47 (CG5486)*	USP47 (50.8)	USP	VDRC: 103743	USP
*scny (CG5505)*	USP36 (38%)	USP	VDRC: 11152	USP
*CYLD (CG5603)*	CYLD (38%)	USP	VDRC: 101414	USP; CAP-Gly
*Usp34/puf (CG5794)*	USP34 (52%)	USP	VDRC: 27517	USP; UBL; ARM
*Usp8 (CG5798)*	USP8 (46%)	USP	VDRC: 107623	USP; MIT; Rhodanese
*Usp12–46 (CG7023)*	USP46 (69%)	USP	VDRC: 100586	USP
*Usp39 (CG7288)*	USP39 (67%)	USP	VDRC: 110535	USP; ZnF-UBP
*PAN2 (CG8232)*	PAN2 (56%)	USP	VDRC: 330711	USP; Exonuclease; WD40
*Usp32 (CG8334)*	USP32 (49%)	USP	VDRC: 18981	USP; EF hand; DUSP
*Usp20–33 (CG8494)*	USP20 (42%)	USP	VDRC: 42609	USP; DUSP; ZnF-UBP
*DUBAI (CG8830)*	USP35 (36%)	USP	VDRC: 28960	USP

FlyBase symbol represents gene names based on the FlyBase database and next to FlyBase symbols, annotation symbols are labeled with CG numbers. Those DUBs which are annotated only with CG-prefixed numbers are labeled with a CG-prefix number only in FlyBase symbol and annotation symbols column. The second column shows the annotated human orthologs of the Drosophila DUBs. Next to the human orthologs, the average identity between Drosophila and human orthologs was labeled in brackets. The Stock centre and Stock ID column contains the ID number and the abbreviated name of the stock centre as follows: the Bloomington Drosophila Stock Center is labeled as Bloom, the Vienna Drosophila Resource Center is denoted to VDRC and the National Institute of Genetics is referred to as NIG-Fly. The last column, depicting the Functional Domains, demonstrates the conserved functional domains where the short names of domains are labeled as follows: *ANK* Ankyrin repeats domain, *ARM* armadillo repeat domain, *CAP-Gly* cytoskeleton-associated protein glycine-rich, *DUSP* domain in USP, *ICP0* Infected cell protein 0, *JAMM* JAB1/MPN/Mov34 metalloenzyme, *MATH* meprin and TRAF homology domain, *MIT* microtubule interacting and transport, *Mit-Mem* mitochondorial membrane, *OTU* ovarian tumour deubiquitinase domain, *RnaseH* Ribonuclease H, *UBA* Ubiquitin-Associated domain, *UBL* Ubiquitin-like domain, *UBP* ubiquitin binding domain, *UBX* Ubiquitin Regulatory X, *UCH* ubiquitin C-terminal hydrolase, *UIM* Ubiquitin Interacting Motif, *USP* Ubiquitin specific protease, *C2H2* Cys2His2-like fold group, *RANBP2* Ran binding protein 2, *Znf* Zinc finger. Asterisks indicate null mutation of the related gene.

1: In case of CG4603, we used the Otu1 UniProt nomenclature as it has no official name in the FlyBase.

Previous studies demonstrated that the loss of *Usp5* causes lethality in the L3 larval stage^26,27^. Furthermore, the *da-Gal4*-driven *Usp5*^*RNAi (17657)*^ exhibited subsequent lethality, but primarily in the pupa stages^27^. In addition, the *da-Gal4*-driven *not*^*RNAi 45775*^ and the *not*^*[5-HA-1189]*^ (*not*^*[P]*^) mutant alleles resulted in L2–L3 lethality in homozygous form (Supplementary Fig. 2 and Supplementary Table 1). Therefore, we investigated the *Usp5*^*1*^*/TM6b GFP* (green fluorescent protein) and *not*^*[P]*^*/TM6b GFP* heterozygous mutant Drosophila stocks and monitored their sensitivity to UV light. Unfortunately, in this case, we did not determine any significant phenotypic changes compared with those in the control (Fig. 1b). These results indicate that several DUBs are required in the UV-induced DSB repair pathway in *D. melanogaster*. Previous research also demonstrated that USP45 plays a role in the UV-induced DNA repair pathway and *USP45*-knockout exhibits hypersensitivity upon UV irradiation^28^. According to our UV screening results, the *Usp16–45* DUB mutant demonstrated the highest sensitivity to UV exposure, which is consistent with the results of previous human cell culture-based research^28^. Interestingly, the *Usp47*^*RNAi (103743)*^ Drosophila strain also exhibited decreased viability, which confirmed a previous study that implicated it in the base excision repair (BER) pathway^29^, whereas Otu1 and Usp14 were found to be involved in DNA double-strand break repair^21,30^.

### Otu1 and Usp5 are involved in the X-ray-induced DNA double-strand break repair

**Figure 2:**
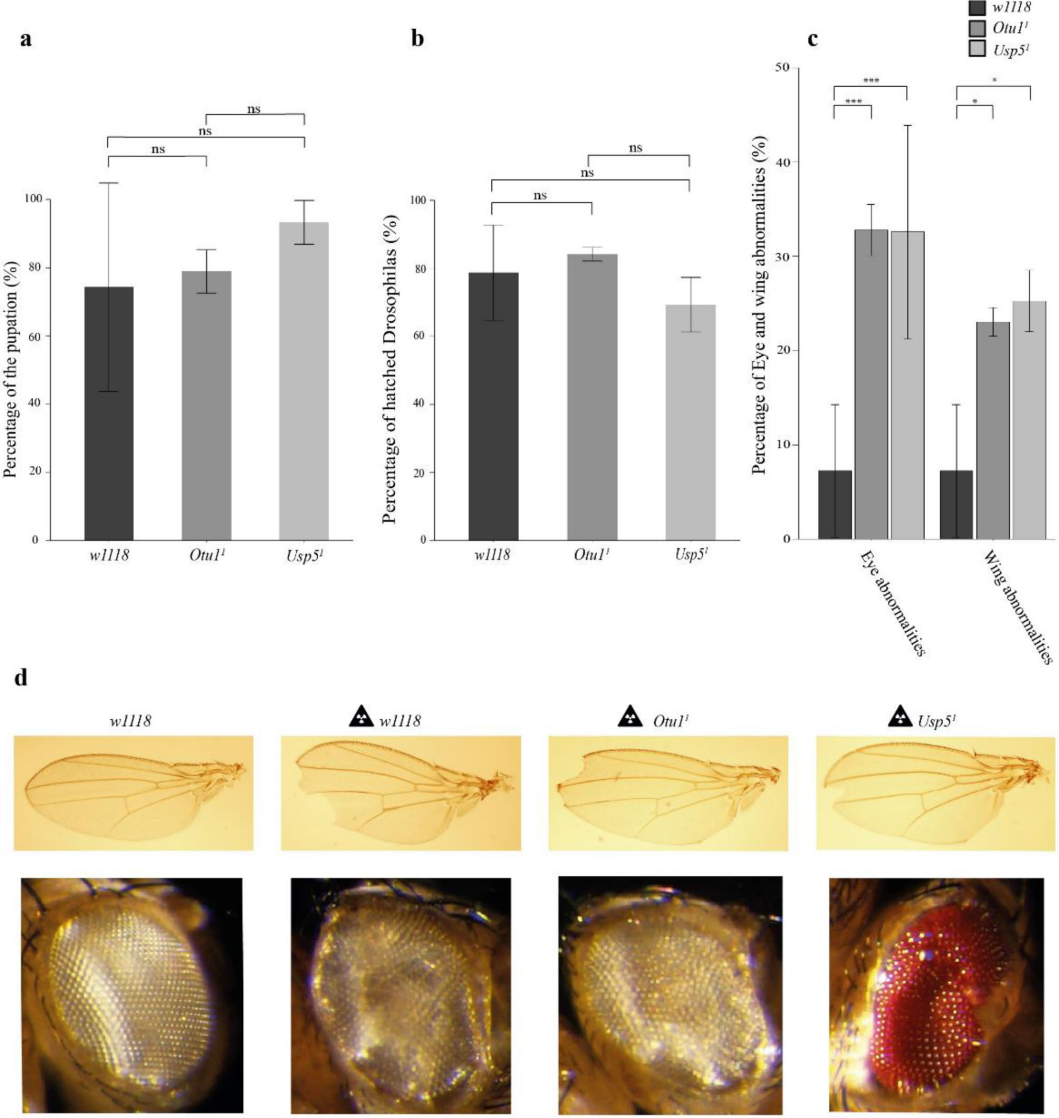
Effect of X-ray irradiation on the homozygous *Otu1*^*1*^ (w^+^; + ;CG4603^Δ101/2^/CG4603^Δ101/2^) mutant and the heterozygous *Usp5*^*1*^ mutant (w^+^; + ;Usp5^1^/TM6b GFP**)** Drosophila. (**a**) and (**b**) L3 DUB mutant larvae were irradiated with 40 Gy X-ray, and the rates of pupation and eclosion were measured (**a** and **b**, respectively). (**c**) The percentage of wing and eye abnormalities was represented. In both the mutants and the control lines, the number of Drosophila with abnormale eye and wing phenotype was compared to the number of the hatched Drosophila which survived the X-ray treatment. (**d**) The detected wing and eye defects in adult DUB mutants are depicted. For the statistical analysis, one-way ANOVA in combination with Tukey’s post-hoc test was used. Significant values are indicated as follows: *P < 0.05, ***P < 0.001, ****P < 0.0001. Data were obtained from three independent experiments.

In the first screening, we identified several DUBs whose functions are essential in the UV-induced DNA repair pathway. In the next step, we evaluated whether these DUBs are also essential for the repair of other types of DNA damage. For this purpose, we applied X-ray irradiation that causes DSBs, and we checked the pupation and eclosion rates of Drosophila harboring DUB mutations. We used *Usp5*^*1*^*/TM6b GFP* and *Otu1*^*1*^ (w^+^;+;*CG4603*^*Δ101*/2^/*CG4603*^*Δ101*/2^) Drosophila mutants which exhibited different sensitivities in response to UV irradiation. The DUB mutant third instar larvae were irradiated with 40 Gray (Gy) X-ray, and after the treatment we evaluated the survival rate of the larvae. We observed that the 40 Gy dose had no significant effect on either the pupation or eclosion rate of the DUB mutant compared with that of controls (Fig. 2a and b and Supplementary Fig. 3). However, following hatching, we detected abnormal eye and wing phenotypes in *Otu1*^*1*^ and *Usp5*^*1*^ mutant flies. The wing and eye abnormalities were also detected in *w1118* flies, but the occurrence of these defects was significantly higher in DUB mutants (Fig. 2c,d). Interestingly, although the X-ray irradiation directly caused DSBs, we found that the *Otu1* mutant flies were more sensitive to UV irradiation than to X-ray exposure compared with the control (Figs. 1a and 2a–c). These results indicated that the loss of *Otu1* resulted in a more severe phenotype in the UV-induced DNA repair pathways than in the X-ray irradiation-induced DSB repair pathway, suggesting a diverse function of Otu1. However, the *Usp5*^*1*^ mutant flies showed no significant differences subsequently to either UV or X-ray irradiation, although the occurrence of eye and wing abnormalities was significantly higher after the ionizing irradiation, indicating the putative regulatory but dispensable role of Usp5 in DSB repair pathways.

### Usp34 is essential for euchromatic site-specific DSB repair in DR-white system

**Figure 3:**
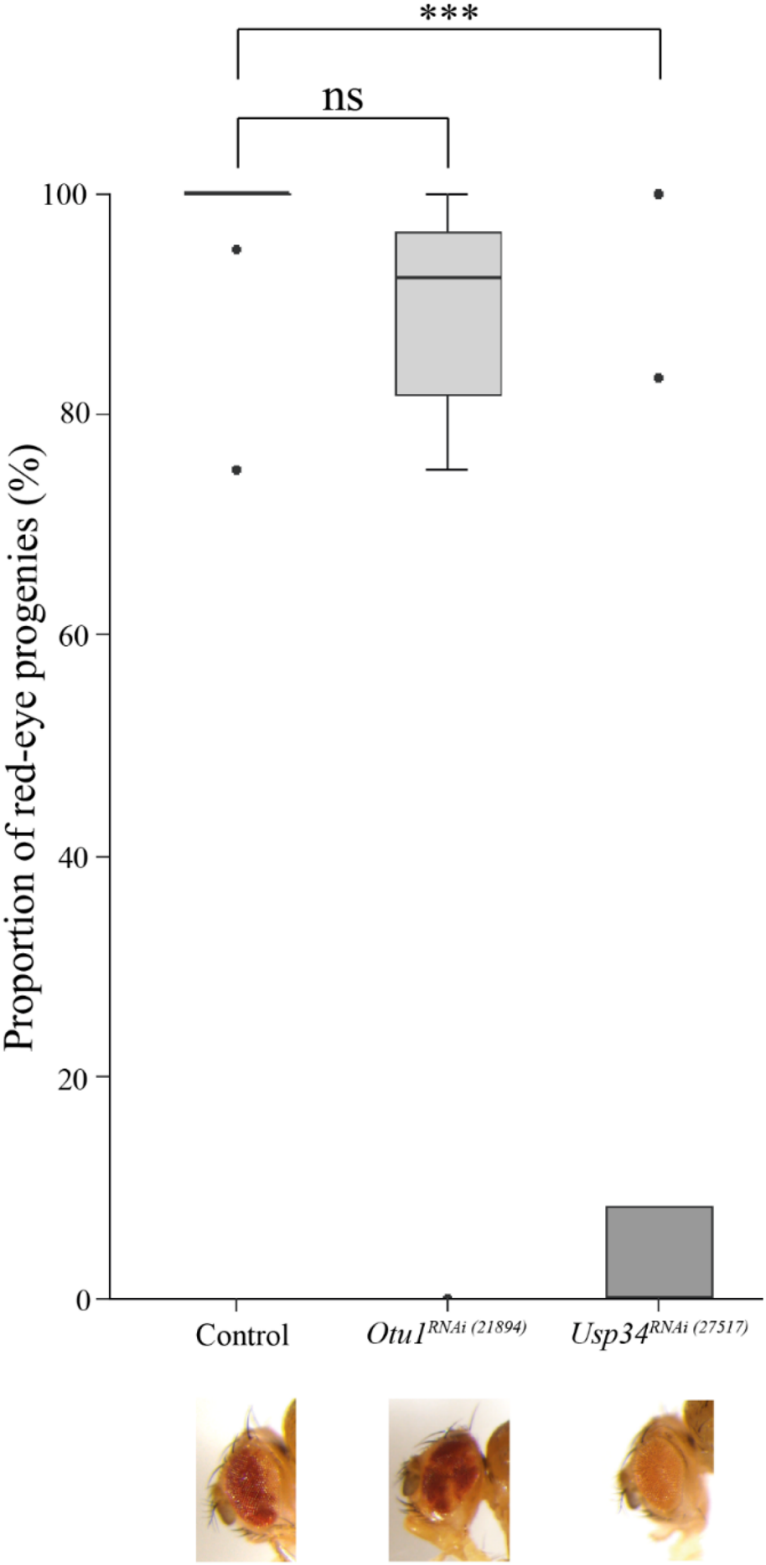
Phenotypic changes were obtained using I-SceI-induced site-specific DSB system in Otu1^RNAi (21894)^ and Usp34^RNAi (27517)^ DUB mutants (DR-white assay). The DR-white assay allows determining the choice of the repair pathway following I-Sce1-induced site-specific DSBs. Otu1 and Usp34 RNAi transgenes were driven by da-GAL4, the L3 larvae were heat-shocked to induce site-specific DSBs in the DR-white transgenes, and the mosaic red-eye phenotype was detected. Representation of the ratio of red-eye phenotype applying the euchromatic DR-white system. In case of *Otu1*^*RNAi (21894)*^ we used heat-shock-induced *yw; DR-white.y* + *(DR-white_2eu_1)/hsp70.HA.I-Sce, Sco; da-GAL4/Otu1*^*RNAi (21894)*^ adult flies and compared the number of flies with red eye to the total number of the flies with the above-described genotype. In case of *Usp34*^*RNAi (27517)*^ we used the same quantification by measuring the red-eye mosaic phenotype. The heat-shock-induced *yw; DR-white.y* + *(DR-white_2eu_1)/hsp70.HA.I-Sce, Sco; da-GAL4/Usp34*^*RNAi (27517),*^ flies were counted and the mosaic red-eye adult flies were compared to the total number of the adult flies with the above-described genotype. For the control, we used y*w; DR-white.y* + *(DR-white_2eu_1)/hsp70.HA.I-Sce, Sco; da-GAL4/*+ Drosophila lines with the same quantification method as was used for *Usp34*^*RNAi (27517)*^ and *Otu1*^*RNAi (21894)*^. For the statistical analysis, Welch's ANOVA in combination with Dunnett's T3 post-hoc test was used. Significant values are indicated as follows: *P < 0.05, **P < 0.01, ****P < 0.0001. Data were obtained from *nine* independent experiments. More details about the DR-white system are described in a previous study^31^.

Our results demonstrated that Otu1 and Usp5 play an indispensable role in the damage-induced DNA repair pathways. DSB formation may also be induced by inaccurately repaired T-T dimers caused by UV exposure. Regarding the cell cycle phase, DSBs can induce either non-homologous end joining (NHEJ) or HR, which are the two predominant DNA double-strand breaks repair (DSBR) pathways. In addition, DUBs have already been shown to be involved in DNA repair pathways through the deubiquitylation of regulatory proteins involved in DDR, NHEJ, or HR. To examine whether Otu1 is in fact required for HR, we used the previously mentioned DR-white assay to investigate the homology-directed repair frequency in the absence of this DUB. The Drosophila DR-white assay was developed by Anthony T. Do and Joseph T. Brooks et al., which allows determining the pathway choice among HR, NHEJ, and SSA during DSBR^31^. This *in vivo* system contains two nonfunctional repeats of the *white* gene, which enclose the *yellow (y*+*)* transgene: (I) the upstream part, referred to as *Sce.white*, and contains I-SceI recognition sequences, and (II) the downstream non-functional *white* repeat, which due to the truncation does not possess either the 5′ and 3′ UTRs or the promoter and transcription start sequences of the *white* gene. The heat-shock-induced expression of I-SceI generates site-specific DSBs at I-SceI recognition sites and activates DSBR. In the progenies, we could monitor the phenotypic changes caused by the activation of the appropriate DNA repair pathway. In the *yw* genetic background, the brown bodies and white eyes indicated no DSB induction, HR, or NHEJ repair. If the more accurate HR repair occurs, the I-SceI recognition site will be repaired from the adjacent *iwhite* gene, resulting in red-eye progenies. The *DR-white* transgenes were integrated in both euchromatic and heterochromatic regions, and therefore, the site-specific DSB repair events can be measured in both regions (Supplementary Fig. 4). In our experiment, we investigated whether the HR-related recombination frequency was affected in the absence of DUBs. For this purpose, we used the *Otu1*^*RNAi (21894)*^ (*yw; DR-white.y*+ *(DR-white_2eu_1)/hsp70.HA.I-Sce, Sco; da-GAL4/Otu1*^*RNAi (21894)*^) and *Usp34*^*RNAi (27517)*^ RNAi stocks (*yw; DR-white.y*+ *(DR-white_2eu_1)/hsp70.HA.I-Sce, Sco; da-GAL4/Usp34*^*RNAi (27517)*^) that also contained the heat-shock-inducible I-SceI-coding gene and the DR-white transgene on the 2^nd^ chromosome. We also applied the *da-GAL4* driver to induce the depletion of *Otu1* and *Usp34*. As a control, we used the y*w; DR-white.y*+ *(DR-white_2eu_1)/hsp70.HA.I-Sce,Sco; da-GAL4/*+ Drosophila strain that contains the same DR-white and I-SceI transgenes on the 2^nd^ chromosome and the *da-GAL4* driver on the 3^rd^ chromosome. The euchromatic DR-white assay showed that the ratio of red-eye offsprings was much lower in the *Usp34*^*RNAi (27517)*^ than in the control strain. However, in the absence of Otu1, we found no significant differences in HR efficiency compared with the control (Fig. 3). These results suggested that Usp34 is an important regulator of HR repair in *D. melanogaster*. Previous research demonstrated that OTU protein family members in human cells can regulate RNF168 activity which together with RNF8 are important regulators of DDR, although the loss of Drosophila Otu1 did not result in any difference in HR efficiency compared with the control. We also did not detect any Drosophila adults with mosaic red-eye color in the heterochromatic DR-white assay in either the control or the RNAi DUB Drosophila strain (data not shown). This result indicated that either I-SceI is not effective in the heterochromatic region with regard to site-specific DSB induction or these DUBs are not essential for repairing heterochromatic DSBs.

## Discussion

Reversible PTMs play pivotal roles during the regulation of DNA repair pathways. Among these PTMs, ubiquitylation is not only involved in controlling the stability of DNA repair proteins, but it also mediates the recruitment of additional repair proteins to the damaged locus. Although several E3 ubiquitin ligases have been shown to be involved in K48- or K63-linked polyubiquitylation of specific DNA repair proteins, the DUBs that remove ubiquitin moieties from DNA repair proteins are poorly characterized^32^. For identifying novel DUBs implicated in DNA repair, we conducted an RNAi screening using specific DUB siRNA-expressing Drosophila strains to explore their function in UV-, X-ray-, and site-specific I-SceI-induced DNA damage. Among the DUB strains we tested, we found that *Usp47*, *Usp34*, *Usp14*, *Otu1*, *Usp16–45*, *Usp5*^*1*^, and *not*^*[P]*^ DUB mutant Drosophila strains exhibited severe UV light-induced phenotype compared with the control. We identified a milder effect in *Usp5*^*1*^ and *not*^*[P]*^ DUB mutants, indicating that these DUBs are required, but probably dispensable for the appropriate repair of UV light-induced DNA damage in *D. melanogaster*. Furthermore, the silencing of *Usp47*, *Usp34*, *Usp4*, and *Otu1* was fatal in these larvae, and *Usp16–45*-silencing resulted in the most robust phenotype alteration compared with the control.

Consistently, earlier studies also revealed that USP45 is important in the UV-induced DNA repair pathways, especially in the deubiquitylation of Excision repair cross-complementation group 1 (ERCC1), which is the subunit of Xeroderma pigmentosum, complementation group F-Excision repair cross-complementation group 1 (XPF-ERCC1) implicated in Transcription-Coupled Nucleotide Excision Repair (TC-NER) regulation^28,33^. These data also supported the sensitivity of our screening in which we identified additional DUBs, including Usp47, Usp34, Usp14, and Otu1, required for the UV-induced DNA damage-related repair processes.

Although the *Usp47* mutant larvae showed less sensitivity to UV irradiation compared with larvae with the loss of other DUBs, Usp47 may affect the survival rate upon UV irradiation through a different pathway. As demonstrated by Parsons et al. that USP47 regulates the level of Pol β in BER, the lower sensitivity of USP47 can be explained by the diverse role of the desired DUBs in the DNA repair pathways^29^. This assumption is further supported by the fact that USP34 and USP14 are also implicated in the regulation of the HR pathway, whereas OTU1/OTUB1 mediates the RNF168-dependent K63-linked polyubiquitylation^17,20,21,30,34^.

All these results are consistent with our findings showing that several DUBs, which we identified in the UV screening, have already been demonstrated to be essential in other DNA repair pathways than NER, emphasizing the specificity of the screening, and that these proteins are in fact essential players in the UV-induced DNA repair pathways.

As a next step, we induced DSBs using ionizing irradiation to explore the role of *Otu1* and *Usp5* in DSB repair. We found that X-ray irradiation did not affect the survival rate of Drosophila larvae in either *Otu1*^*1*^ or *Usp5*^*1*^ DUB mutant Drosophila, but eye and wing abnormalities were detected at a significantly higher rate than that in the control. These results suggest that the absence of *Otu1* and *Usp5* does not play an essential role in the X-ray-induced DNA repair pathway, but it probably decreases the efficiency of the repair itself. Our hypothesis is that it delays the repair in fast-dividing diploid tissues, which resulted in milder phenotypic abnormalities, but does not affect the overall viability of the flies. The Drosophila Otu1 and its human homology (YOD1) are poorly characterized in DNA repair pathways, but previous studies have demonstrated that both the OTU family members of DUBs such as OTUB1 and USP5 play a role in DSB repair, especially in HR^21,35^. It was also demonstrated that in the absence of USP5, γH2AX removal is delayed from the DSB sites in HR and the improper HR increases the sensitivity against DSB-induced agents^35,36^. This finding also supports our results that in the absence of Usp5 and Otu1, DNA repair is still functional, but less effective in DSB repair. In our system, the inappropriate HR repair might result in the appearance of phenotypic alterations detected in Drosophila tissues. To verify this phenomenon, we used the DR-white *D. melanogaster* assay which is an I-SceI meganuclease-based site-specific DSB-inducer system. This experimental setup depends on the *white* transgene that causes mosaic red–white eye color in Drosophila when DSBR occurs. Using this system, we detected significantly lower red-eye-color-containing progenies in the case of *Usp34* silencing, whereas the loss of *Otu1* did not cause any significant difference compared with the control. Earlier, it was also demonstrated that OTU family members of proteins (OTUB1 and OTUB2) can differently influence the pathway choice. The depletion of OTUB2 suppresses the HR pathway through the RNF8-mediated ubiquitylation; in contrast, the depletion of OTUB1 caused increased HR through the inhibition of ATM^21^. Furthermore, USP34 can stabilize RNF168 through deubiquitylation, and in the absence of USP34, the level of RNF168 is decreased^17^. This effect can influence the pathway choice through the accumulation of 53BP1 at DSB sites^37^. We also observed that the absence of DUBs can influence the pathway choice in Drosophila differently. We showed that HR was not suppressed with the decreased level of *Otu1*, but the silencing of *Usp34* can inhibit HR, indicating a diverse role of the two DUBs in the HR pathways as well.

In our experimental setup, we used various types of DNA-damaging agents, such as UV irradiation, ionizing radiation, and oxidative damage, which can cause different types of DNA lesions. For repairing these DNA lesions, cells have evolved various well regulated DNA repair pathways. Since the activation of an inappropriately chosen DNA repair circuit can be harmful for the cells, these pathways are tightly regulated by PTMs, such as ubiquitylation, and therefore, DUBs can be key regulators of these processes. Our screening demonstrated the diverse function and importance of several DUBs in the UV- and X-ray irradiation-induced DSB repair pathways. We observed that the absence of Usp47, Usp34, Usp14, Otu1, and Usp45 can affect the survival rate of Drosophila after UV irradiation, indicating an important role of these DUBs in DNA repair. We also demonstrated that Otu1, Usp5, and Usp34 play a regulatory, but dispensable role in DSBR. These results emphasize the importance of using multicellular in vivo model system to study the function of DUBs implicated in DNA repair, and the phenotypic changes observed in our system can provide a more sensitive method for identifying novel DUBs implicated in NER and DSBR.

## Materials and methods

### Identification of DUBs in *D. melanogaster*

In the first step, the sequences of already known yeast and human DUBs were used for the identification of Drosophila DUBs, which have been reviewed by Amerik et al. and Nijman et al.^24,25^. Moreover, a preliminary bioinformatics analysis was performed to identify the DUB-sequences in Drosophila based on the yeast sequence homology. Sequences were downloaded from NCBI, UniProt, and FlyBase, and for identifying the homology sequences, BLAST program was used. EMBOSS Needle Pairwise Sequence Alignment program of EMBL's European Bioinformatics Institute (EMBL-EBI's) allowed comparing the protein sequences, and EMBL-EBI's Interpro Scan program ensured to identify domains in the proteins. This complex identification system enabled the accurate identification of the various DUBs in Drosophila.

### UV irradiation screening

The *da-GAL4* driver stock was used to diminish the levels of *Usp47*, *Usp34*, *Usp14*, *Otu1*, and *Usp16–45* using transgenic RNAi Drosophila stocks. We applied the *da-GAL4* driver in the homozygous form on the 2nd chromosome, and the RNAi transgene was inserted into the 3rd chromosome. All of the RNAi stocks used in this study were kindly provided by Péter Deák (Table 1). The early L3 larvae were UV-irradiated with a dose of 15 mJ/cm^2^. For measuring the survival rate of the UV-treated larvae, the animals were collected and maintained on standard Drosophila medium. Data were obtained from three independent biological replicates, and in each experiment, 50 larvae were exposed to UV irradiation. The *w1118* stock was used as a control (Supplementary Fig. 1).

### Drosophila stocks and mutation generation

The fly stocks were maintained on standard medium at 25 °C. The transgenic RNA interference and the P element insertion-containing lines were obtained from Bloomington Drosophila Stock Center, Vienna Drosophila Resource Center, and NIG-Fly National Institute of Genetics. The RNAi lines with Stock IDs are depicted in Table 1. We used the following three null mutant Drosophila lines:

#### *usp5*^*1*^ mutant allele carrying Drosophila line

Using P element remobilization technique, the imprecise excision of *P{EPgy2}EY20760* generated 275-bp deletion, eliminating the catalytic histidine box and UBA2 domain coding sequence. The deletion causes L3 lethality in homozygous form. More information about the stock is available in Kovács et al.^27^. In our UV screening, the mutant allele was balanced with TM6b GFP.

#### PCR *not*^*[P]*^ (*not*^*[5-HA-1189]*^) mutation carrying Drosophila line

*not*^*[5-HA-1189]*^ mutation causes L2 and L3 lethality in the homozygous form (Supplementary Table 1). We measured the expression of *not* genes using semi-quantitative reverse transcription–polymerase chain reaction (RT-PCR). We did not detect any expression of *not* genes in the *not*^*[5-HA-1189]*^ homozygous mutant Drosophila larvae compared with the control (Supplementary Fig. 2). The *not*^*[5-HA-1189]*^-containing stock is available in the Drosophila Genetic Resource Center Kyoto with the Stock ID number 125130.

#### *Otu1*^*1*^ (*CG4603*^*Δ101*/2^) mutant allele carrying Drosophila line

**Figure 4:**
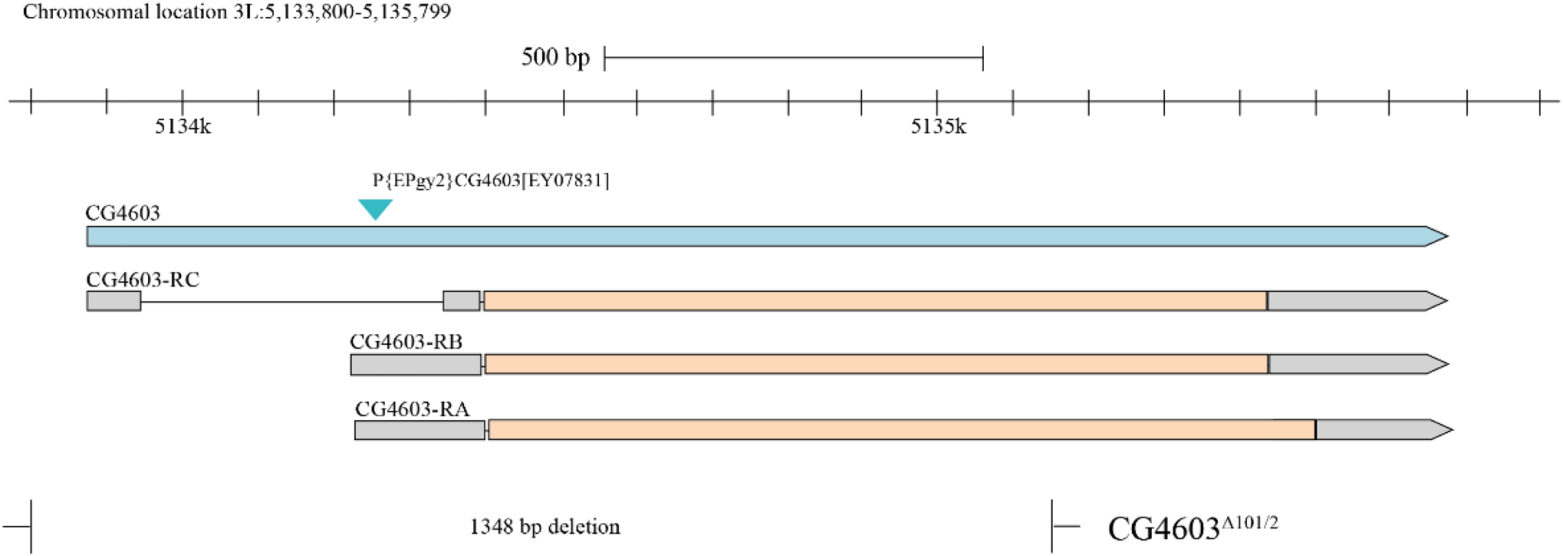
Schematic representation of CG4603 P element insertion and CG4603^Δ101/2^ deletion allele. The blue triangle represents the P element insertion, and the broken line shows the deleted segment of CG4603 gene. CG4603-RC, CG4603-RB, and CG4603-RA are the transcripts of CG4603. Figure was prepared on the basis of the FlyBase Genome Browser.

For CG4603^Δ101/2^ deletion, the P element remobilization technique was used. The imprecise excision of P{EPgy2}CG4603(EY07831) generated the *CG4603*^*Δ101*/2^ deletion allele containing 1348-bp deletion which eliminates two-third part of the gene (Fig. 4). We did not detect significant lethality in the homozygous form compared with the control (Supplementary Table 1).

### Semi-quantitative RT-PCR

Total RNA was extracted from 10 larvae using the Tri Reagent extraction kit (Sigma-Aldrich, USA). RNA samples were treated with RQ1 RNase-free DNase (Promega Corporation, USA). The Thermo Fisher Scientific cDNA synthesis kit was used for reverse transcription, according to the manufacturer’s instruction. Samples were normalized to the *rpL17A* level using the following primers: *RpL17A* forward 5′-GAGCCAAGAACCTGTACG-3′ and reverse 5′-CAGGCATGACCTTCTTCC-3′. Gene expressions were determined using 20-25-cycle PCRs with exon-specific primers. The RT-PCR was quantified by using Fiji Image J (Supplementary Fig. 2).

### X-ray irradiation

The null mutant *Otu1*^*1*^ and *Usp5*^*1*^ Drosophila stocks were kindly provided by Péter Deák. Because of the strong L3 lethality, in the case of *Usp5*^*1*^, we used *w;*+*; Usp5*^*1*^*/TM6b GFP* Drosophila larvae for X-ray irradiation. The *Otu1*^*1*^* (w;*+*; CG4603*^*Δ101/2*^*/CG4603*^*Δ101/2*^*)* null mutant Drosophila was used as the homozygous mutant form during X-ray irradiation. *w1118* stock was used as a control. In case of control (*w1118)* and DUB mutant Drosophila [*Otu1*^*1*^* (w;*+*; CG4603*^*Δ101/2*^*/CG4603*^*Δ101/2*^*) and Usp5*^*1*^, we used *w;*+*; Usp5*^*1*^*/TM6b GFP*], the early L3 stage larvae were collected and inserted into the sample holder of the X-ray machine and then exposed to an X-ray dose of 40 Gy (Supplementary Fig. 3). Subsequently to X-ray irradiation, 100 irradiated larvae were collected in each experiment (three independent experiments were performed) and maintained on standard Drosophila medium.

### Drosophila DR-white assay

For the euchromatic DR-white assay, yw; DR-white.y+ (DR-white_2eu_2)/CyO; da-GAL4 females were crossed either with yw; hsp70.HA.I-Sce,Sco/CyO; UAS-RNAi CG4603 or with yw; hsp70.HA.I-Sce,Sco/CyO; UAS-RNAi Usp34 males. The embryos from these crosses yw; DR-white.y+ (DR-white_2eu_1)/hsp70.HA.I-Sce,Sco; da-GAL4/Otu1^RNAi (21894)^ and DR-white.y+ (DR-white_2eu_1)/hsp70.HA.I-Sce,Sco; da-GAL4/UAS-RNAi Usp34 were heat-shocked at 38 °C for 1 h. For the mosaic red-eye phenotype quantification, we used heat-shock-induced yw; DR-white.y+ (DR-white_2eu_1)/hsp70.HA.I-Sce, Sco; da-GAL4/Otu1^RNAi (21894)^ adult flies and we compared the number of red-eye flies to the total number of flies with the above-described genotype. In case of Usp34^RNAi (27517)^, we used the same quantification method by measuring the red-eye mosaic phenotype. The heat-shock-induced yw; DR-white.y+ (DR-white_2eu_1)/hsp70.HA.I-Sce, Sco; da-GAL4/Usp34^RNAi (27517)^ were counted and the mosaic red-eye adult flies were compared to the total number of the adult flies with the above-described genotype. For the control, we used yw; DR-white.y+ (DR-white_2eu_1)/hsp70.HA.I-Sce, Sco; da-GAL4/+ Drosophila lines with the same quantification method as was used in case of Usp34^RNAi (27517)^ and Otu1^RNAi (21894)^.

For the heterochromatic DR-white assay, we used yw; DR-white.y+ (DR-white_2het_2)/CyO; da-GAL4 females, which were crossed with transgenic yw; hsp70.HA.I-Sce, Sco/CyO; UAS-RNAi CG4603 or yw; hsp70.HA.I-Sce, Sco/CyO; UAS-RNAi Usp34 Drosophila males. The original Drosophila DR-white stocks (yw; DR-white.y+ (DR-white_2eu_2)/CyO and yw; DR-white.y+ (DR-white_2het_2)/CyO) and the yw; hsp70.HA.I-Sce,Sco/CyO Drosophila stock were kindly provided by Jeannine R. LaRocque and Anthony T. Do. More details about the transgenic DR-white stock are described in Janssen et al.^38^. The RNAi Drosophila stocks were kindly provided by Péter Deák. For the DR-white assay, nine independent experiments were performed.

## Supplementary Information

Supplementary Information 1.

Supplementary Information 2.
