# Supplementary material for: Usp5, Usp34, and Otu1 deubiquitylases mediate DNA repair in Drosophila melanogaster
Source: Sci Rep. 2022 Apr 7;12:5870. doi: 10.1038/s41598-022-09703-x (PMC8990000; doi:10.1038/s41598-022-09703-x)
Supplement: Supplementary file 1 — Supplementary Information 1. [file 41598_2022_9703_MOESM1_ESM.pdf]

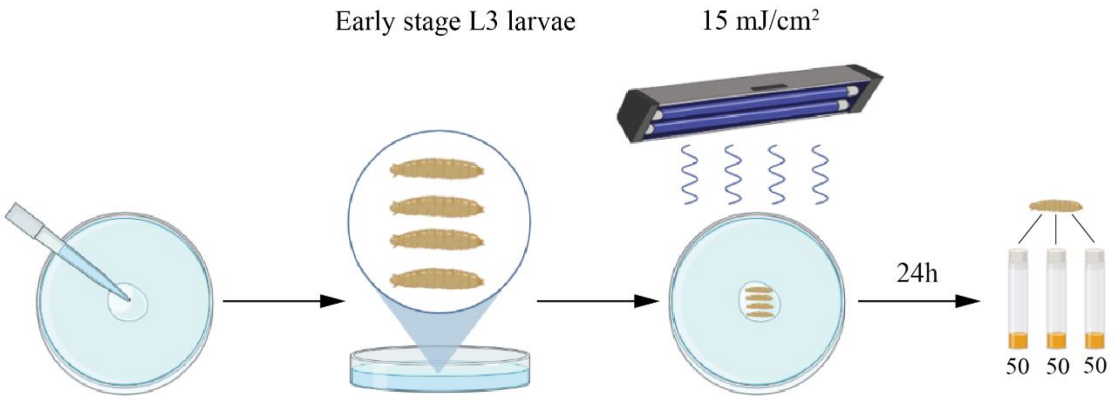

Supplementary Figure 1.: The schematic representation of the applied UV irradiation.

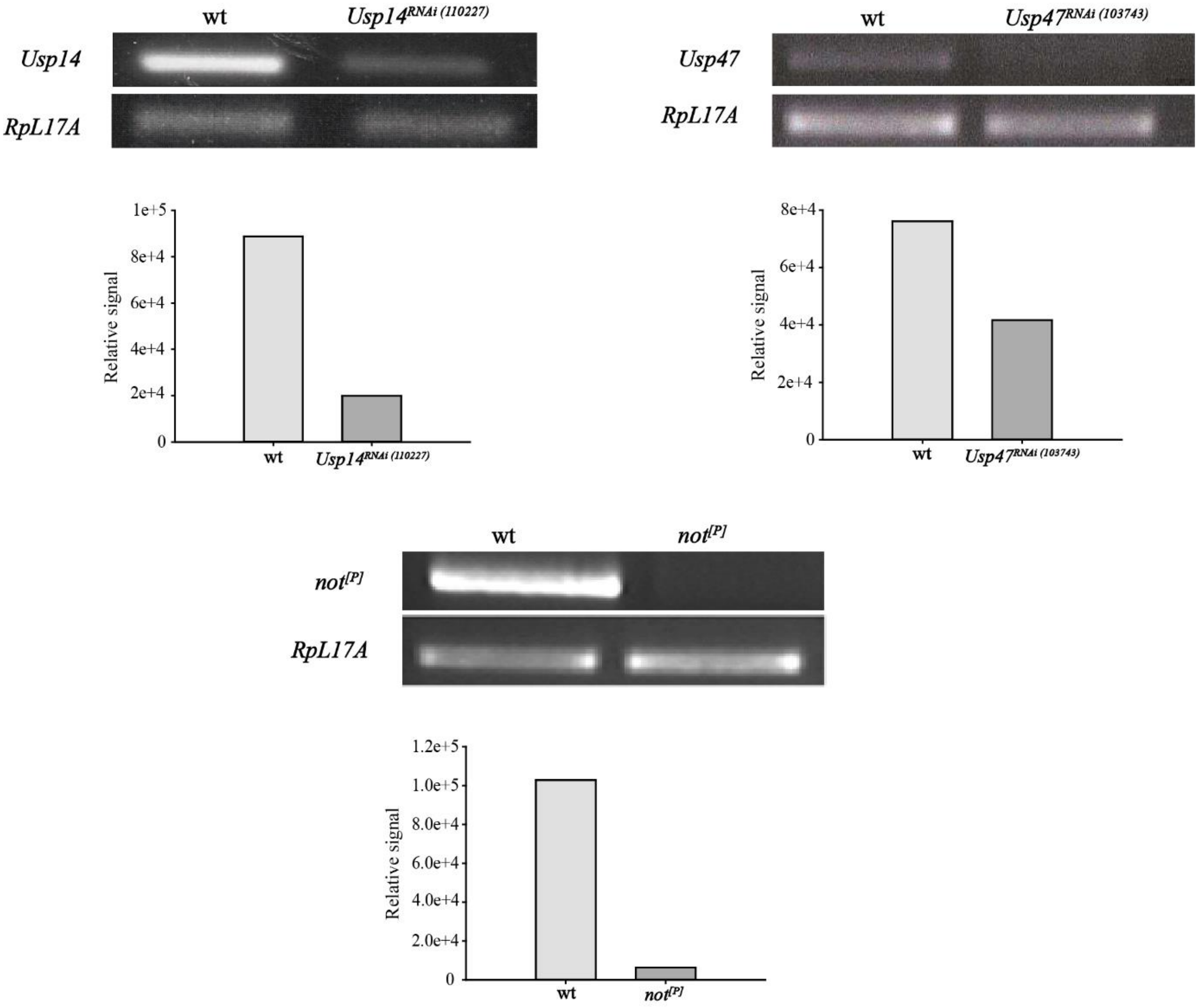

Supplementary Figure 2.: Semi-quantitative RT-PCR of wild type (WT) and the used RNAi stocks and *not*[5-HA-1189] mutant Drosophila line. We used rpL17 as a loading control. The RT-PCR was quantified using Fiji Image J. On the y axis, the relative pixel intensities of the signals are represented.

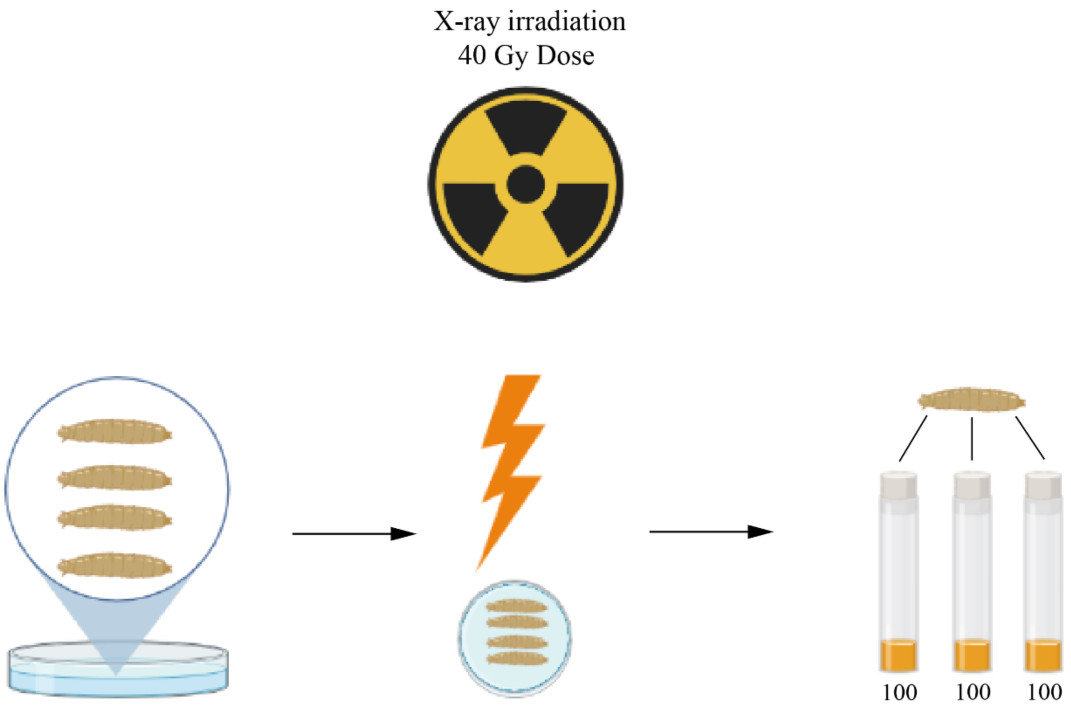

Supplementary Figure 3.: The schematic representation of the applied X-ray irradiation.

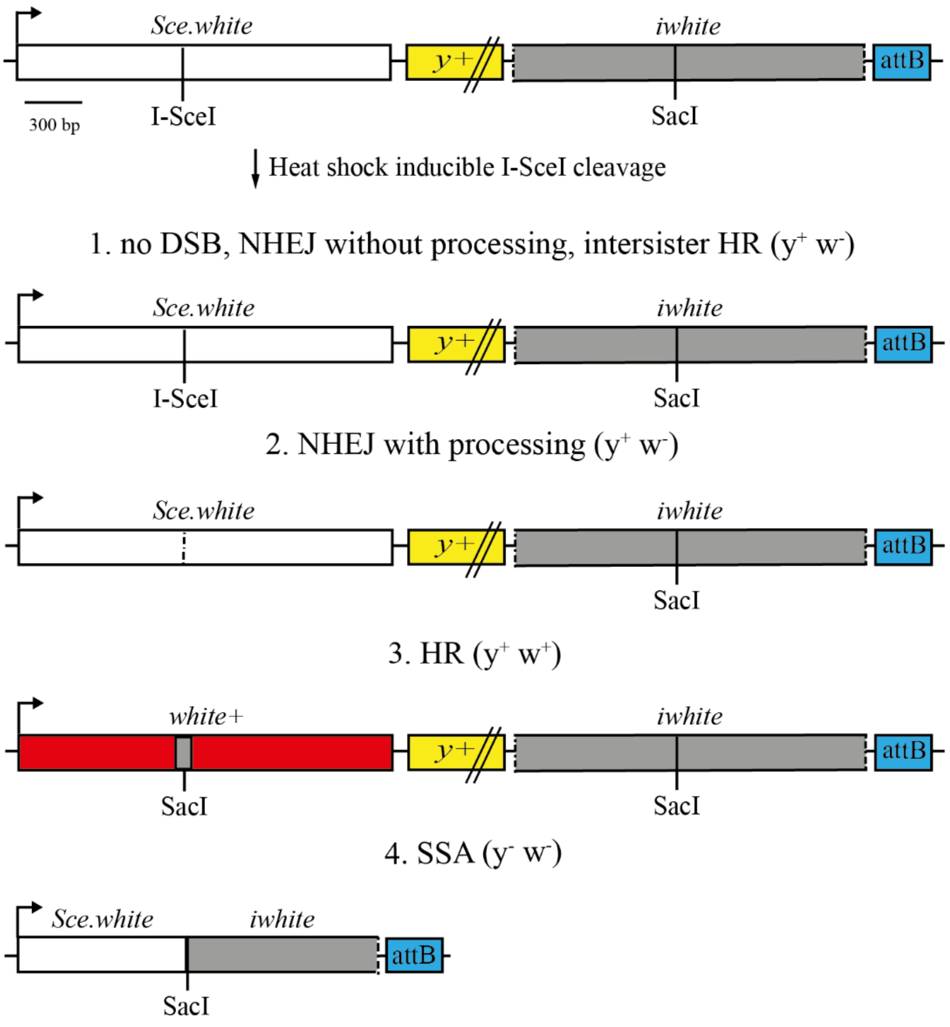

Supplementary Figure 4.:

Supplementary Figure 4.: Schematic representation of DR-white system. The figure made based on Anthony T. Do and Joseph T. Brooks et al.[31]. More details are in the article.

	Lethalphase				
	L2 larvae	L3 larvae	Pupa	Pharate adult	Viable
w1118				11%	89%
not$^{[P]}$	81%	19%			
Otu^{1}				22%	78%

Supplementary Table 1.: Lethality phase of *not*$^{5\text{-}HA\text{-}1189}$ and *CG4603*$^{\Delta 101/2}$ null mutant Drosophila lines.
