# Supplementary material for: Usp5, Usp34, and Otu1 deubiquitylases mediate DNA repair in Drosophila melanogaster
Source: Sci Rep. 2022 Apr 7;12:5870. doi: 10.1038/s41598-022-09703-x (PMC8990000; doi:10.1038/s41598-022-09703-x)
Supplement: Supplementary file 2 — Supplementary Information 2. [file 41598_2022_9703_MOESM2_ESM.pdf]

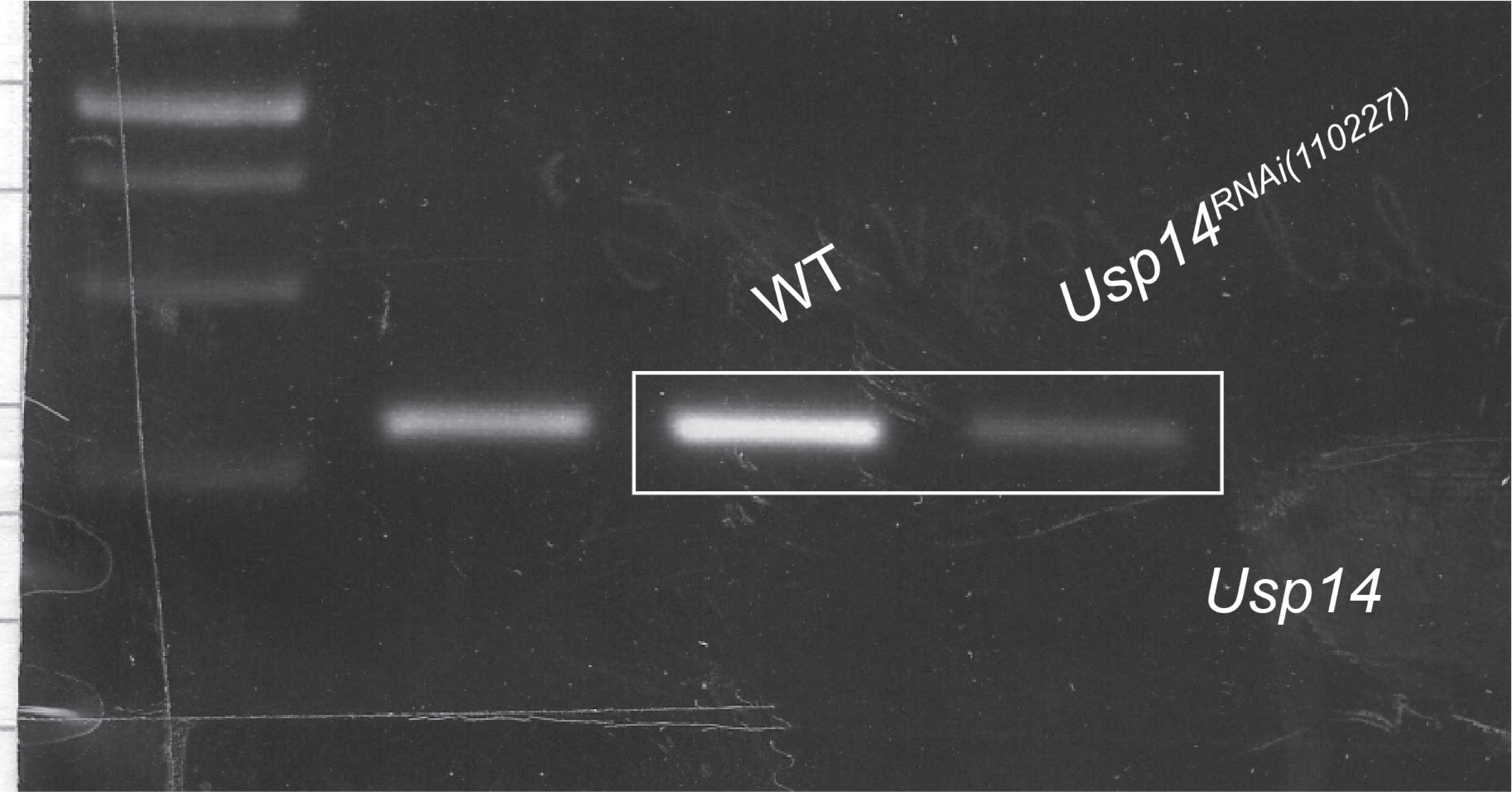
WT
Usp14^RNAi(110227)
Usp14

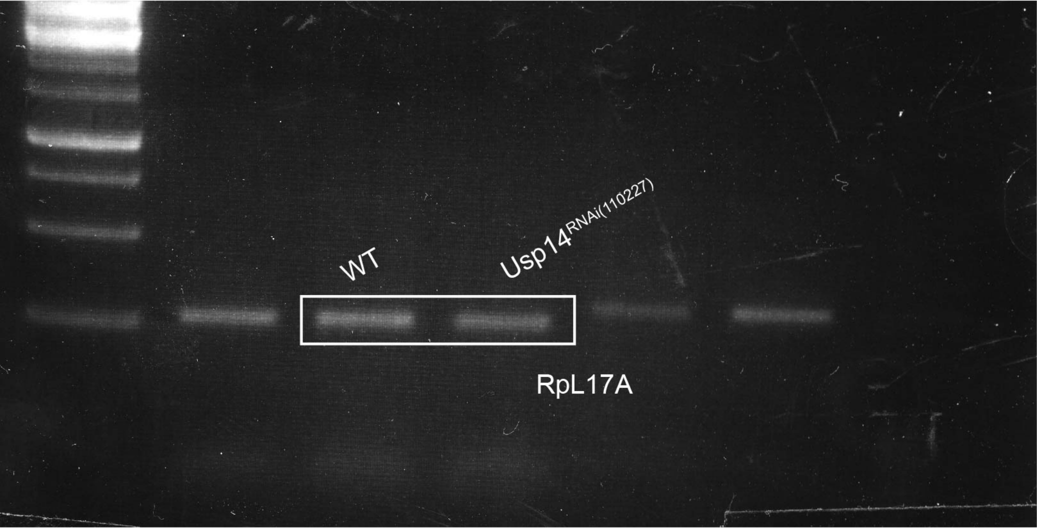
WT
Usp14^RNAi(110227)
RpL17A

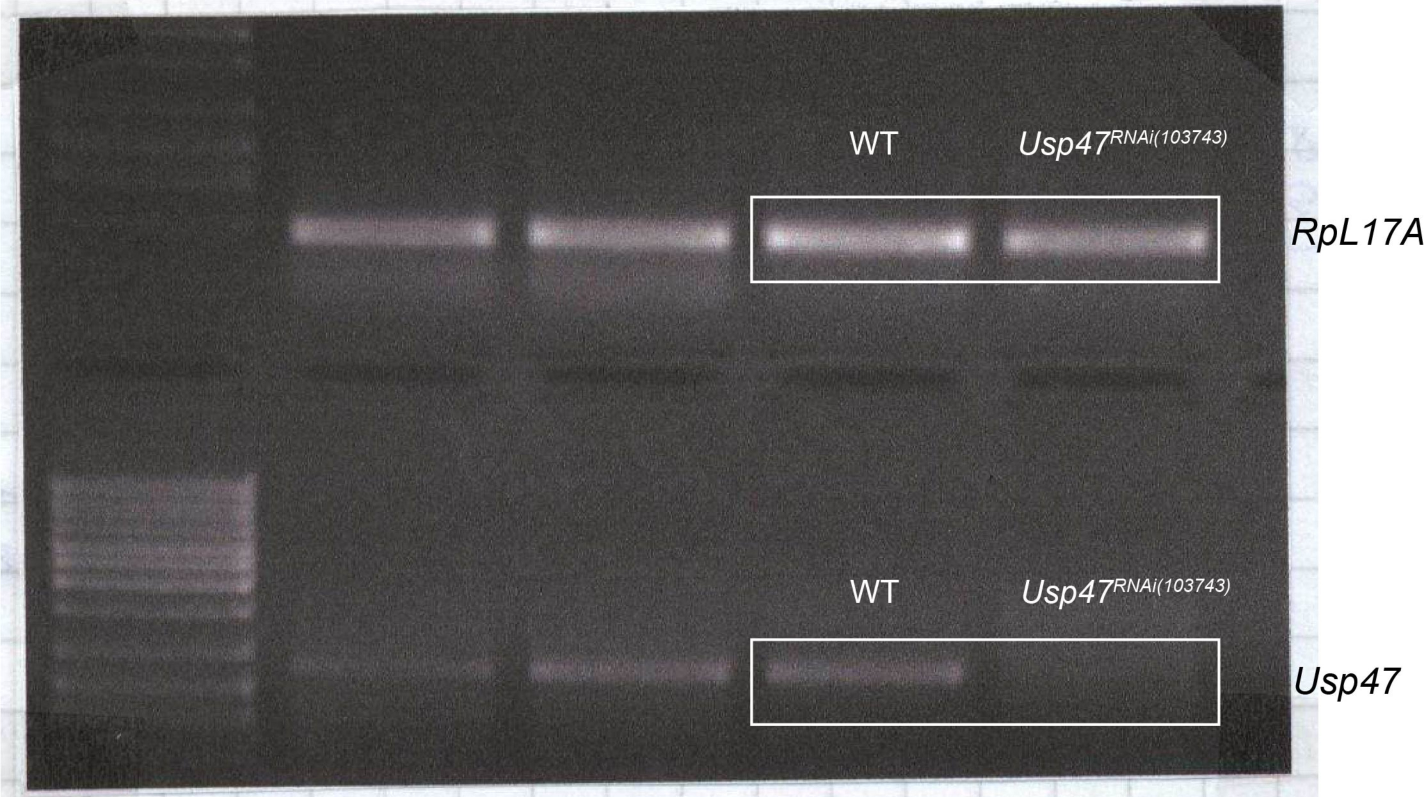
WT
$Usp47^{RNAi(103743)}$
RpL17A
WT
$Usp47^{RNAi(103743)}$
Usp47

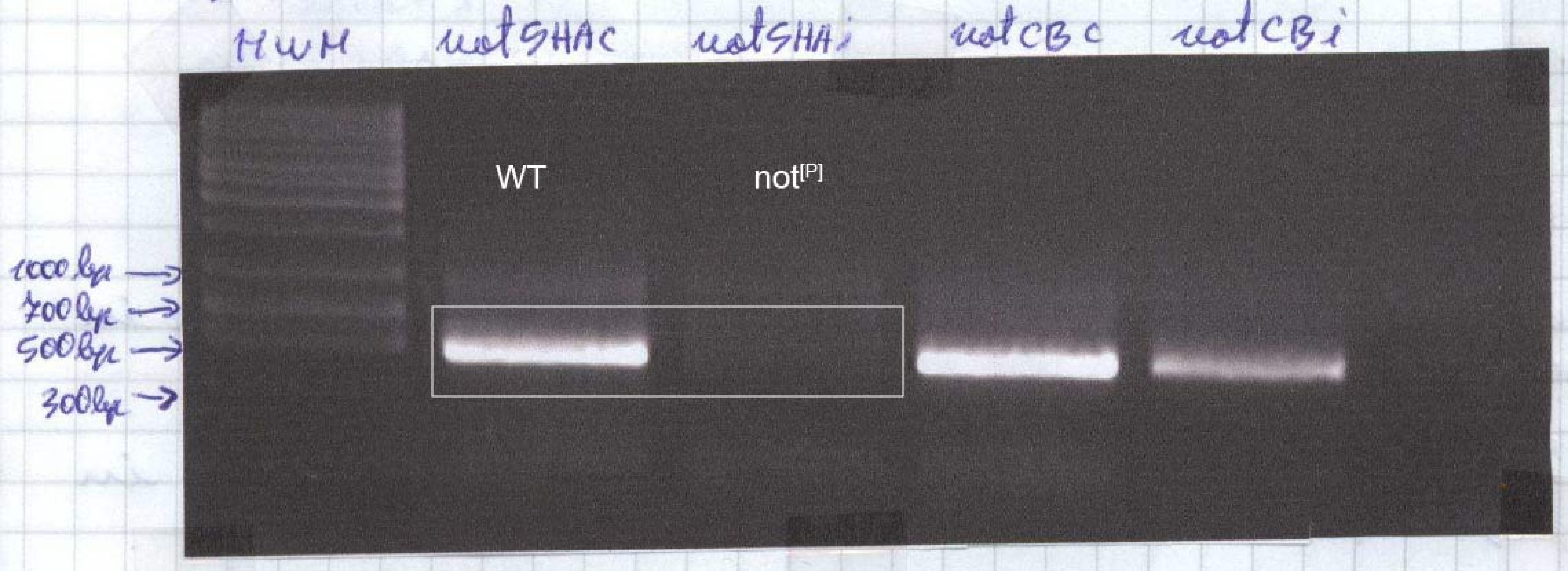
MWM
notSHAC
notSHAi
notCBC
notCBi
WT
not[P]
1000 bp
700 bp
500 bp
300 bp

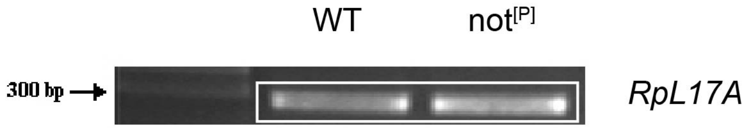
WT
not[P]
300 bp
RpL17A
